# Tunable electromagnetically induced transparency in coupled three-dimensional split-ring-resonator metamaterials

**DOI:** 10.1038/srep20801

**Published:** 2016-02-09

**Authors:** Song Han, Longqing Cong, Hai Lin, Boxun Xiao, Helin Yang, Ranjan Singh

**Affiliations:** 1 College of physical science and technology, Central China Normal University, Wuhan 430079, China; 2 Division of Physics and Applied Physics, School of Physical and Mathematical Sciences, Nanyang Technological University, Singapore 637371, Singapore; 3 Centre for Disruptive Photonic Technologies, School of Physical and Mathematical Sciences, Nanyang Technological University, Singapore 637371, Singapore; 4 Engineering Geophysical Research Center, Yangtze University, Jingzhou 434023, China

## Abstract

Metamaterials have recently enabled coupling induced transparency due to interference effects in coupled subwavelength resonators. In this work, we present a three dimensional (3-D) metamaterial design with six-fold rotational symmetry that shows electromagnetically induced transparency with a strong polarization dependence to the incident electromagnetic wave due to the ultra-sharp resonance line width as a result of interaction between the constituent meta-atoms. However, when the six-fold rotationally symmetric unit cell design was re-arranged into a fourfold rotational symmetry, we observed the excitation of a polarization insensitive dual-band transparency. Thus, the 3-D split-ring resonators allow new schemes to observe single and multi-band classical analogues of electromagnetically induced transparencies that has huge potential applications in slowing down light, sensing modalities, and filtering functionalities either in the passive mode or the active mode where such effects could be tuned by integrating materials with dynamic properties.

Several fascinating properties of metamaterials occur due to electromagnetic coherent coupling between the induced oscillating currents at desired resonance frequencies. Electric as well as magnetic dipolar and high-order multipolar coupling are the commonly employed strategies to induce interference effects in subwavelength plasmonic and metamaterial resonators. These coupling effects between individual constituents play dominant roles in determining the optical properties of metamaterials. In nanostructures, a canonical method to harvest higher-order multipole is to construct plasmonic oligomers, such as disk/ring nanostructures^2^,^3^,^4^, nanoparticle dimers^5^,^6^, trimers^7^, tetramers^8^,^9^,^10^, and some other high-order clusters^11^,^12^,^13^,^14^,^15^,^16^. A hybridization process occurs when these nanoparticle clusters are excited by the incident light. It is the excitation of the multipoles that would induce a destructive interference (anti-bonding effect), which is a dominating approach to observe effects such as classical analog of electromagnetically induced transparency (EIT) phenomenon and the related Fano resonance phenomena^4^,^5^,^6^,^7^,^8^,^9^,^10^,^11^,^12^,^13^,^14^,^15^,^16^,^17^,^18^. Alternatively, the anti-bonding effect between the induced electric dipole and the toroidal dipole has been used to observe the non-radiative resonant transparency^19^. Such a resonant transparency is non-trivial with the extremely high-*Q* resonances due to the low scattering of the electromagnetic field.

In this work, we exhibit the metamaterial coupling induced transparency in three-dimensional (3-D) split-ring resonators (SRRs) system that consists of three SRRs in a unit cell. We analyzed the transverse and longitudinal coupling in this system to understand the coherent coupling mechanism in the proposed structure. Detailed numerical simulations and an analytical coupled oscillator model were applied to reproduce the transparency spectra that would enhance the understanding of the underlying mechanism. The transparency window revealed a strong polarization-dependence behavior that reveals the switch-on and switch-off states with orthogonal polarization excitations. By re-arranging the polarization sensitive six-fold rotationally symmetric unit cells in a four-fold rotational symmetry configuration, we obtained a polarization insensitive transparency feature that exhibits dual-mode transparency windows.

## Results

**Figure 1:**
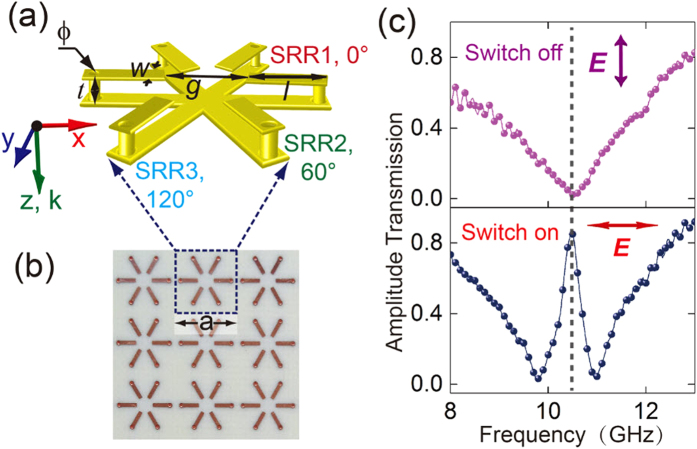
Metamaterial design and the measured spectra. (**a**) The unit cell consists of three SRRs, which is named as SRR1 (0°), SRR2 (60°) and SRR3 (120°), respectively. The specific geometrical parameters of each SRR are *w* = 0.5 mm, *l* = 2.5 mm, *g* = 2 mm, and ϕ = 0.4 mm. (**b**) Top view of the experimental sample. The SRRs are supported by a dielectric substrate (Rogers RO4003) with the geometric dimensions of *a* = 8 mm, thickness *t* = 0.813 mm, and permittivity *ɛ* = 3.55 with loss tangent of 0.0027. (**c**) The measured transmission spectra that reveal the on and off states for the transparency window. The arrows represent the incident polarization of the wave.

We designed the 3-D metamaterial with a unit cell as shown in Fig. 1(a), where each SRR has a split gap on the top panel and a continuous wire in the bottom. In order to connect the top and bottom arms, two vertical metallic cylindrical pillars were fabricated to pierce through dielectric layer to form a three-dimensional SRR. The metal used in the design is Copper. It is well known that an oscillating current loop would be excited as the fundamental inductive-capacitive (LC) resonance mode by the incident electric field that polarized along the split gap or magnetic field that penetrates through the SRR. Under the 3-D configuration, the electric and magnetic fields would excite the mode in phase and we would consider electric field for simplicity. As shown in Fig. 1, the proposed metallic unit cell has six-fold (C6) rotational symmetry. However, this C6 symmetric unit cell with square periodicity on the dielectric substrate (C4 symmetry leads to the polarization-dependent property of the metamaterial (see Supplementary materials for details). Therefore, we define the horizontal polarized incidence as 0° (180°), i.e. the **E**-field is along *x*-direction, and the vertical polarized incidence as 90°, i.e. the **E**-field is along *y*-direction. At 90° polarized incidence, a broad resonance in the transmission spectrum is observed which is attributed to the fundamental resonance mode as shown in Fig. 1(c). However, when the polarization angle of incident light is rotated to 0°, a sharp transparency window with the transmission peak at 10.264 GHz is switched on.

**Figure 2:**
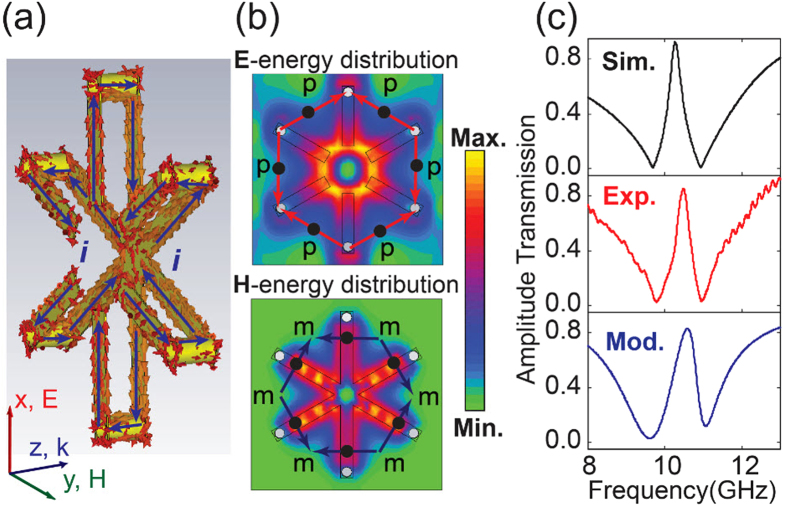
(**a**) The simulated surface currents at the transparent frequency, the blue arrows represent the currents on the front and back metallic strips, respectively. (**b**) The simulated **E**-energy and **H**-energy distribution where the equivalent electric dipoles (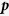) and magnetic dipoles (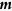) are artificially plotted according to the surface currents. (**c**) The simulated, measured and analytically calculated transmission spectra. For the analytical fit, we used the parameters of ω_0_ = 9.68 GHz, δ = 0.584 GHz, γ_1_ = 2.439 GHz, γ_2_ = 0.14 GHz, Ω = 1.406 GHz.

In order to interpret the mechanism of the induced transparency window, we plot the simulated surface currents on the metallic structure as shown in Fig. 2(a). It is clearly observed that the oscillating currents are distributed on all the three SRRs for 0° polarized incidence at 10.264 GHz which interferes to form the transparency window in the spectrum. Since all the three SRRs are conductively connected through the bottom arms, the oscillating currents can be excited and induced in every SRR which gives rise to conductive coupling resulting in spectrum sharp transparency window. For the intermediate electric field polarization, the resonance is dominated by SRR1 when the angle lies between −30° to 30°. Therefore, SRR1 acts as a bright mode and SRR2, SRR3 are dark mode for this range of angles. Similarly, SRR2 dominates the resonance when the incident polarization angle lies between 30° to 90°, and SRR3, when the range is 90° to 150° (see Supplementary materials for details).

The oscillating currents on the metallic strips is equivalent to electric dipoles 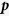, and the loop currents induce magnetic dipoles 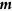 as revealed in Fig 2(b). The electric-/magnetic-energy distributions at the transparent frequency presents hexapolar distribution feature that originates from the LC resonance nature of the structure: the electric energy accumulates at the gap of the SRRs while the magnetic energy is confined at the bottom strips of the SRRs. The electric (magnetic) dipoles plotted in Fig 2(b) present hybridized configuration, where the anti-bonding effect emerges when two oppositely arranged dipoles oscillate out of phase. The out of phase oscillating dipoles would destructively interfere which would tailor the resonant transparency band^1^. The out-of-phase dipoles can be modeled as coupled oscillators^19^,^20^,^21^,^22^:


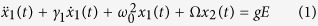



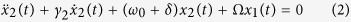


where 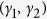 and 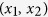 are the damping and the resonant amplitude of two oscillators, respectively. Oscillator 1 couples with the external field (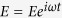) through a coupling strength of *g* and oscillator 2 is a non-resonant mode with respect to the incident excitation, which can be coupled to the resonant mode (oscillator 1) through the near-filed (conductive) coupling. The coupling between the two oscillators is described by the coupling strength of Ω. Considering that the radiative loss could be altered by connecting the SRRs together, we introduce a detuning factor *δ* that represents the frequency difference between the transparency frequency and the resonance frequency of oscillator 1. After solving the above coupled equations (1) and (2) with the displacements vectors expressed as 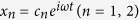, and using the approximation of 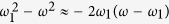
21, the transmission as a function of frequency is obtained as


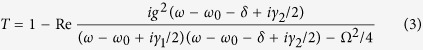


Here we use the scattering parameters of an electric current sheet and the relation of *T* = 1−*R*^22^. The analytically fitted curve using equation 3 reveals a good agreement with the numerical simulation and the experimental results as shown in Fig. 2(c), which reflects the validity of the oscillator model.

**Figure 3:**
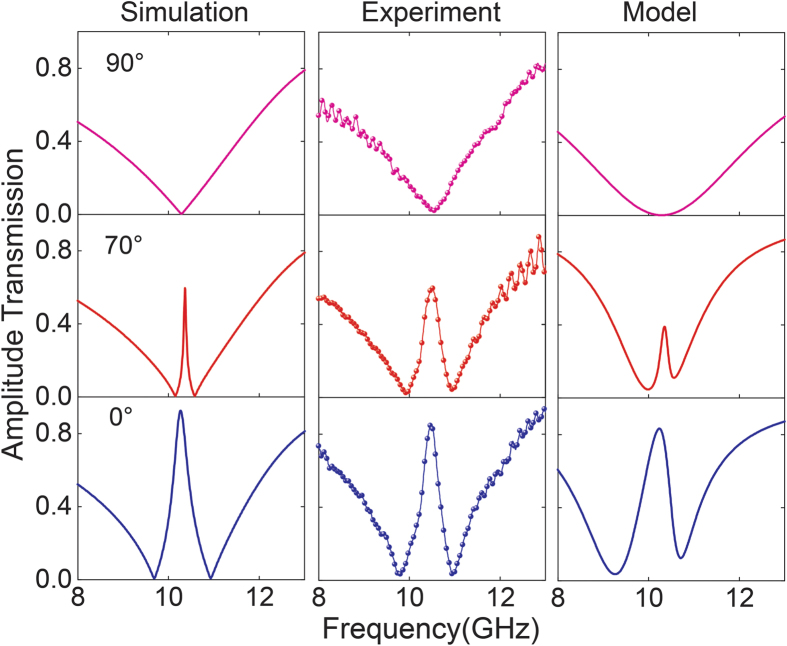
The simulated, measured and analytically modelled transmission spectra at different incident polarization angles.

Since the unit cell is polarization-dependent due to the six-fold rotational symmetry (C6), the metamaterial shows an anisotropic behavior. We therefore investigated the transmission behavior of the 3-D metamaterial at varied incident polarization angles. As shown in Fig. 3, the corresponding results at various incident polarization vectors clearly reveal that the amplitude of the transparent window is gradually modulated as the polarization angle is varied from 0° to 90°. Simulation and experiment results reveal an excellent agreement by comparing the spectra. In addition, by tailoring parameters (γ_1_, γ_2_), ω_0_, δ, and Ω in the coupling model discussed above (see Supplementary materials for details), the modulation behavior of transmission spectra was reproduced in the third column shown in Fig. 3. The measured, simulated and the analytically calculated data reveal the gradual modulation of the transparency window from “on” to “off” state with varied polarization vector direction. According to the coupling model, the radiative loss γ_1_, the detuning δ and the coupling strength Ω between two oscillators are tuned while rotating the incident polarization vector angle from 0° to 90°, where the coupling strength Ω gradually decreases. For the case when the transparency phenomena disappears (90° polarized incidence), Ω is equal to zero which indicates the dependence of coupling strength Ω on the polarization angles that causes the modulation of the transparency band.

**Figure 4:**
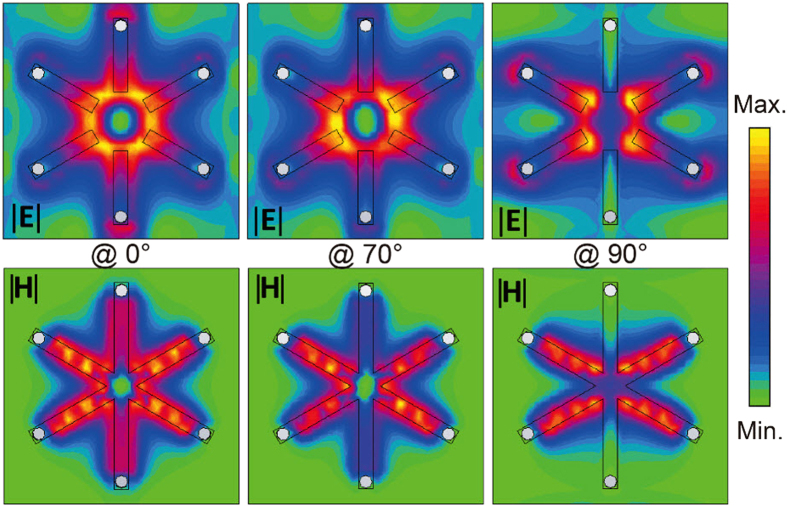
The electric and magnetic field energy distribution at the incident polarization angle of 0°, 70° and 90°, (see Supplementary materials for details).

To visualize the interplay of the scattered near-fields under different incident polarization vectors, we simulated the electric and magnetic energy distributions for varied incident polarization vector as shown in Fig. 4. As we can observe, the electric and magnetic energy is distributed on the three SRRs due to their near-field interaction that results in the transparency window at cases of 0° and 70° polarization and the intensity reveals the modulation of the transparency while the field distribution is only concentrated on SRR2 and SRR3 at 90° polarization. In fact, SRR1 dominates the resonance at 0° polarization incidence where SRR1 gets excited and couples to SRR2 and SRR3 thereby forming anti-bonding dipoles that gives rise to the transparency. With the rotation of the excitation vector to 70°, the resonance is gradually dominated by SRR2 so that SRR2 couples with SRR1 and SRR3, and thereby give rise to transparency induced by near-field anti-bonding effect. However, the electric and magnetic energy distribution on SRR1 disappears when the incident polarization angle is 90°. Under this situation, SRR1 is completely uncoupled to external excitation field. In addition, SRR1 is also uncoupled to SRR2 and SRR3 where we can treat SRR2 and SRR3 as an effective SRR that is perpendicular to SRR1 so that the metamaterial shows the opacity at the 90° incident polarization angle. By rotating the incident polarization vector, both the electric and magnetic energy distributions demonstrate that the near-field interaction in three SRRs dampens gradually, i.e. the coupling strength Ω in coupled oscillator model becomes weaker, which leads to the modulation of the transparency window.

## Discussion

**Figure 5:**
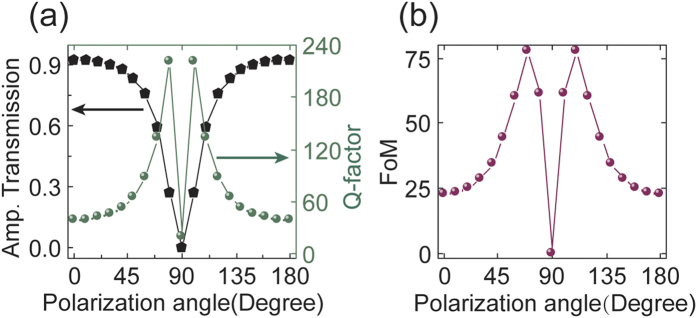
(**a**) Amplitude transmission and the *Q*-factor and (**b**) the FoM of the transparency band at different incident polarization angle.

The transparency window reveals a very sharp line shape which offers the avenue for applications in light-matter interactive systems such as a sensing or a slow light device. In order to give a numerical description of the transparency in terms of the resonance linewidth and its intensity, we show the *Q*-factor and the figure of merit (FoM) obtained through rigorous simulations in Fig. 5. From the figures, we can clearly observe that the amplitude of the transparency window is modulated while rotating the polarization vector and at the same time, the quality factor (*Q*) also changes, as shown in Fig. 5(a). In order to find the trade-off between the *Q* factor and the amplitude of the transparency window, the FoM is defined as 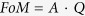, where *A* is the transmission amplitude and *Q* is the quality factor of the transparency window^23^. At around 60° (120°) of the polarization vector, we can observe the optimal FoM value that attains a high value of 77 (see in Fig. 5(b)) with the *Q* factor of 130.3 and amplitude of 0.595 (see in Fig. 5(a)). Such a high value of FoM indicates that the proposed metamaterial can be applied as high *Q*-factor tunable filter and ultrasensitive sensing devices^22^,^23^,^24^,^25^,^26^.

**Figure 6:**
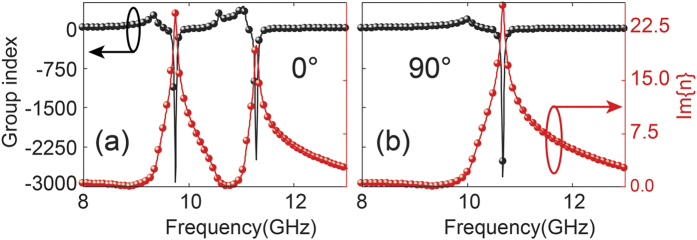
The real part of the group index (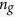) and the imaginary part of the index of refraction [Im(*n*)] as a function of frequency. The incident polarization angle is (**a**) 0° and (**b**) 90°.

In order to clearly understand the slow light effect of the proposed metamaterial, the group index as a function of frequency, i.e., 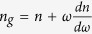, where *n* is the effective refractive index (see Supplementary Materials for details) of the metamaterial and ω is the frequency of light, can be used to depict this slow light property. Figure 6(a) presents the calculated group index (*n*_*g*_) of the proposed metamaterial at the polarization angle of 0°, where the real part of the group index is observed to reveal a large value (up to 427) within the frequency regime of the transparent window. The time delay is calculated as 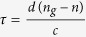, where *d* is the thickness of the substrate (Rogers RO4003) and *c* is the speed of light in vacuum. Therefore, the calculated time delay is up to 1.19 ns within the transparent band. The giant group index at the transparent range shows that the proposed metamaterial can slow down the propagating EM waves efficiently^22^. For comparison, the calculated group index (*n*_*g*_) at 90° polarization angle is also shown in Fig. 6(b), where the group index disappears and reveals a giant dip at the transmission minima. According to the time delay calculation, the slow light effect disappears at 90° polarization angle. The imaginary part of the refraction index is also calculated as shown in Fig. 6(a), where almost a near zero value is observed at the transparent frequency. However, we see two very strong peaks at the transmission dips, which implies that the incident light was transmitted with smaller loss in the transparency range and strongly scattered and absorbed by the material at the two transmission dips.

**Figure 7:**
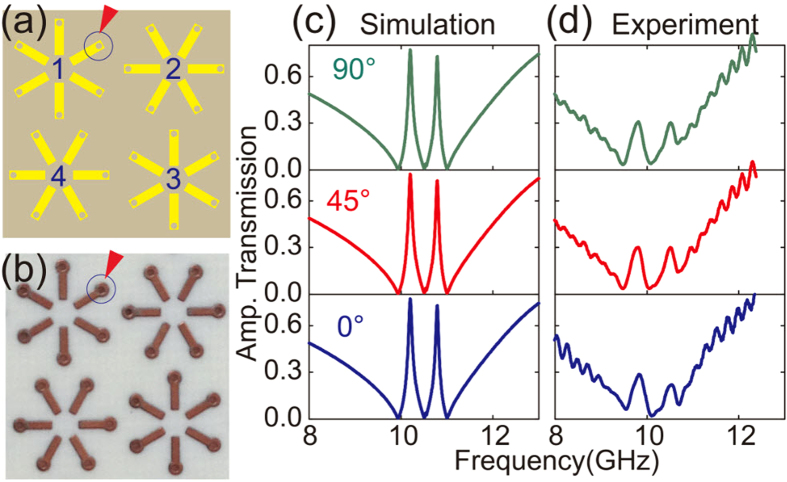
Four-fold (C4) rotationally symmetric unit cells with orthogonally twisted meta-atoms 1, 2, 3 and 4 are shown in (**a**) artistic and (**b**) fabricated version, respectively. Simulated (**c**) and experimental (**d**) results present dual-mode polarization-independent transparencies.

As we have demonstrated, the proposed 3-D design can switch the transparency window from “on” to “off” state with varied polarization vectors that manifests the feature of polarization dependent EIT behavior. However, the polarization insensitivity could also be realized by re-designing the unit cell to preserve the intrinsic C4 symmetry. We introduce four resonators in a unit cell with a C4 symmetry^26^,^27^,^28^,^29^ as shown in Fig. 7(a,b). We experimentally as well as numerically probed the transmission spectra under such a configuration which is displayed in Fig. 7(c,d). The simulated and the corresponding measured transmission spectra exhibit similar transmission behavior with vertically (0°), diagonally (45°) and horizontally (90°) polarized incident waves, demonstrating the polarization independence. Additionally, the transmission spectra shows a pronounced dual-mode transparency window. The dual-mode transparency could be understood if we re-examine the hybrid unit cell of the metamaterial structure. The new pattern consists of a layout where the diagonal resonator pair in the unit cell is mutually twisted by 90°. In this new design, the two transparent windows originate from the nearest neighbor coupling among the four meta-atoms in the unit cell. As we discussed previously, the transparency windows of meta-atom 1 and 3 are switched on with high *Q*-factor at 0° polarization incidence, but those of meta-atom 2 and 4 are switched off which leads to a low *Q*-factor resonance. With the intra meta-atom coupling under the 0° polarization excitation, mode interference between the meta-atoms results in two transparency windows in the spectra as shown in Fig. 7(c). This implies that the diagonally arranged metamaterial support destructive interference through the nearest neighbor coupling^30^,^31^,^32^. As a result, the new arrangement of meta-atoms (C4 symmetry) demonstrates a dual-mode transparency. For the case of 90° incidence, the meta-atom 2 and 4 are switched on and meta-atom 1 and 3 are switched off, which would support the same interference spectra. For the incident angle from 0° to 45°, meta-atom 1 and 3 support low-*Q* EIT and meta-atom 2 and 4 support high-Q EIT. However, they could interfere with each other through nearest neighbor coupling. Therefore, the high-Q transparent spectrum (atoms 2 and 4) overlaps with the low-Q spectrum (atoms 1 and 3) and gives rise to the dual-mode EIT phenomena. Similar arguments could be applied for the incident angles varying from 45° to 90°. Some mismatch between the measured and the simulated data appear in Fig. 7, which mainly originates from the low quality of perforated joints as indicated by the red triangle in Fig. 7(a).

In summary, we have experimentally and numerically provided a novel design scheme using three dimensional metamaterial structures that support classical analog of electromagnetically induced transparency and slow light behavior. The spectral features and the underlying physics are explained by using the anti-bonding mode of hybridized electric-/magnetic-dipoles. The coupling between hybridized dipoles can be understood in terms of the coupled oscillator model, which shows the destructive interference based transparency in the chosen metamaterial design. The six-fold rotationally symmetric structure demonstrated in this work showed polarization dependent modulation of the metamaterial induced transparency window. However, by re-arranging the meta-atoms into a four-fold rotational symmetry in the unit cell, we could achieve polarization independent dual-mode transparency. We believe that the three dimensional metamaterial design presented in this work will play an important role in extending the multifunctional device applications, such as optical switches, high-*Q* tunable band-pass single mode and multimode filters, frequency selective devices, optical modulators, and ultrasensitive sensors^33^,^34^,^35^.

## Methods

The full-wave numerical simulation software CST microwave studio was employed to analyze the spectral response. Open boundary conditions were set along the light propagating direction (z-direction), and unit cell boundary conditions were applied at the x-y plane. After optimizing the parameters in simulations, experimental samples with overall dimension of 200 × 200 mm^2^ were fabricated using the printed circuit board (PCB) technique, whose images of top and bottom panel are shown in Fig. 1(b). The spectral responses were measured by two linearly polarized horn antennas used as an emitter and a receiver. A vector network analyzer (Agilent N8362B) was employed to store and retrieve data.

## Additional Information

**How to cite this article**: Han, S. *et al.* Tunable electromagnetically induced transparency in coupled three-dimensional split-ring-resonator metamaterials. *Sci. Rep.* **6**, 20801; doi: 10.1038/srep20801 (2016).

## Supplementary Material

Supplementary Information
