# Supplementary material for: Tunable electromagnetically induced transparency in coupled three-dimensional split-ring-resonator metamaterials
Source: Sci Rep. 2016 Feb 9;6:20801. doi: 10.1038/srep20801 (PMC4746651; doi:10.1038/srep20801)
Supplement: Supplementary Information [file srep20801-s1.pdf]

Supplementary Material for **"Tunable electromagnetically induced transparency in coupled three-dimensional split-ring-resonator metamaterials"**

Song Han[1], Longqing Cong[2, 3], Hai Lin[1], Boxun Xiao[4], Helin Yang[1, *], and Ranjan Singh[2, 3]

[1]College of physical science and technology, Central China Normal University, Wuhan 430079, China

[2]Division of Physics and Applied Physics, School of Physical and Mathematical Sciences, Nanyang Technological University, Singapore 637371, Singapore

[3]Centre for Disruptive Photonic Technologies, School of Physical and Mathematical Sciences, Nanyang Technological University, Singapore 637371, Singapore

[4]Engineering Geophysical Research Center, Yangtze University, Jingzhou 434023, China

1. The L-C resonant behavior of a single SRR

The Split-ring-resonator (SRR) is the most common resonant element of MMs, as shown in Fig. S1(a). The SRR can be equivalent to inductor-capacitor (L-C) circuit, the metal loops function as inductors and the gap between the metal strips as capacitors of capacitance C. For the sub-wavelength MMs, the electric field penetrates the whole volume of the metallic structure, thereby resulting in equivalent dipolar response under the collective effect of induced currents. That is, the incident electric field (*x*-direction) perpendicular to the gap can excite loop currents (***i***) on the SRR and thereby induce magnetic dipole ($\vec{m}$), as shown in Fig. S1(a). And because of the currents are not uniform distribution on the front and back strips, the oscillating currents can be equivalent to an oscillating electric dipole along the incident electric field direction. The surface electric currents, electric energy and magnetic energy distribution at the resonance are simulated and plotted in Fig. S1(b), where we observe the surface currents coincide with the description of Fig. S1(a). Moreover, the electric energy concentrates around the gap and the magnetic energy concentrates on the back metallic strip, which demonstrates the SRR is surely support inductor-capacitor (L-C) resonance.

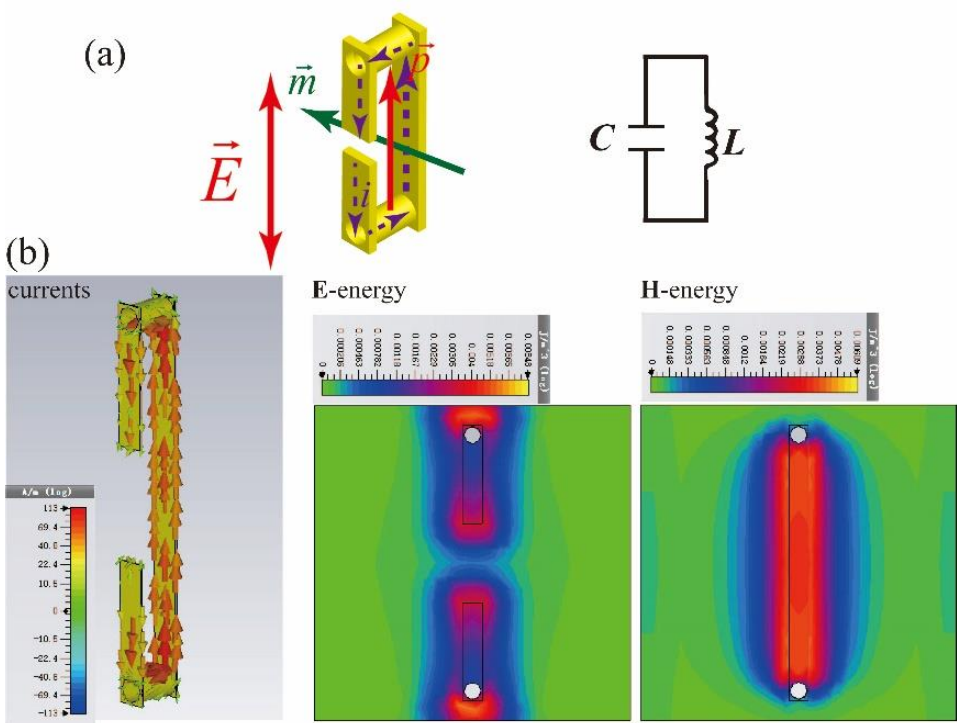

Figure S1. The resonant behavior of a single SRR. (a) The incident excitation induces oscillating currents that can be equivalent to magnetic dipole and electric dipole. (b) The surface electric currents, electric energy and magnetic energy distribution at the resonance.

2. The transmission spectra of single SRR, double SRRs, and three SRR system

For the anisotropic configuration of the metamaterial array, the resonant behavior is tunable for different incident polarization angle. The transmission spectra through rigorous simulations are exhibited in Figure S2. Fig. S2(a) shows the transmission curves for 0° arranged SRR1, 60° arranged SRR2, and 120° arranged SRR2 with the incident polarization angle in steps of 10°. Fig. S2(b) show the transmission curves of the combined SRR. Fig. S2(c) shows the transmission curves of our proposed structure.

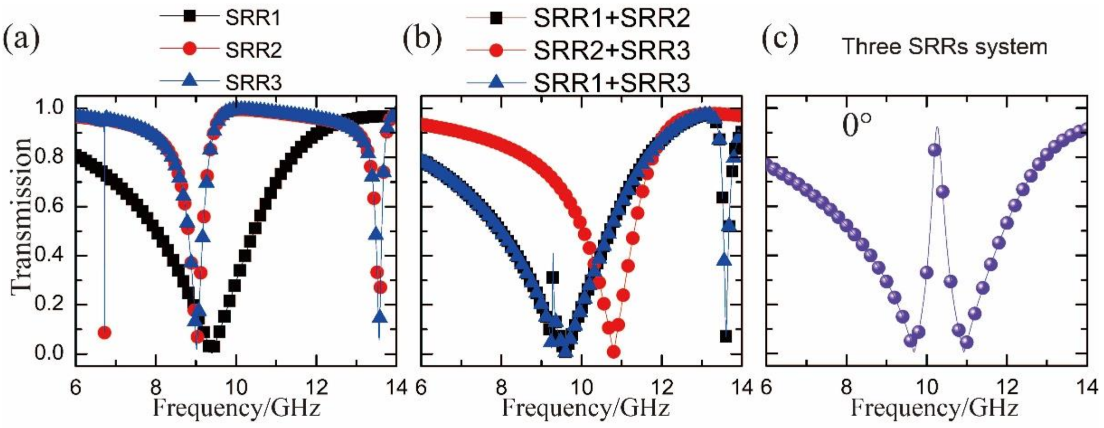

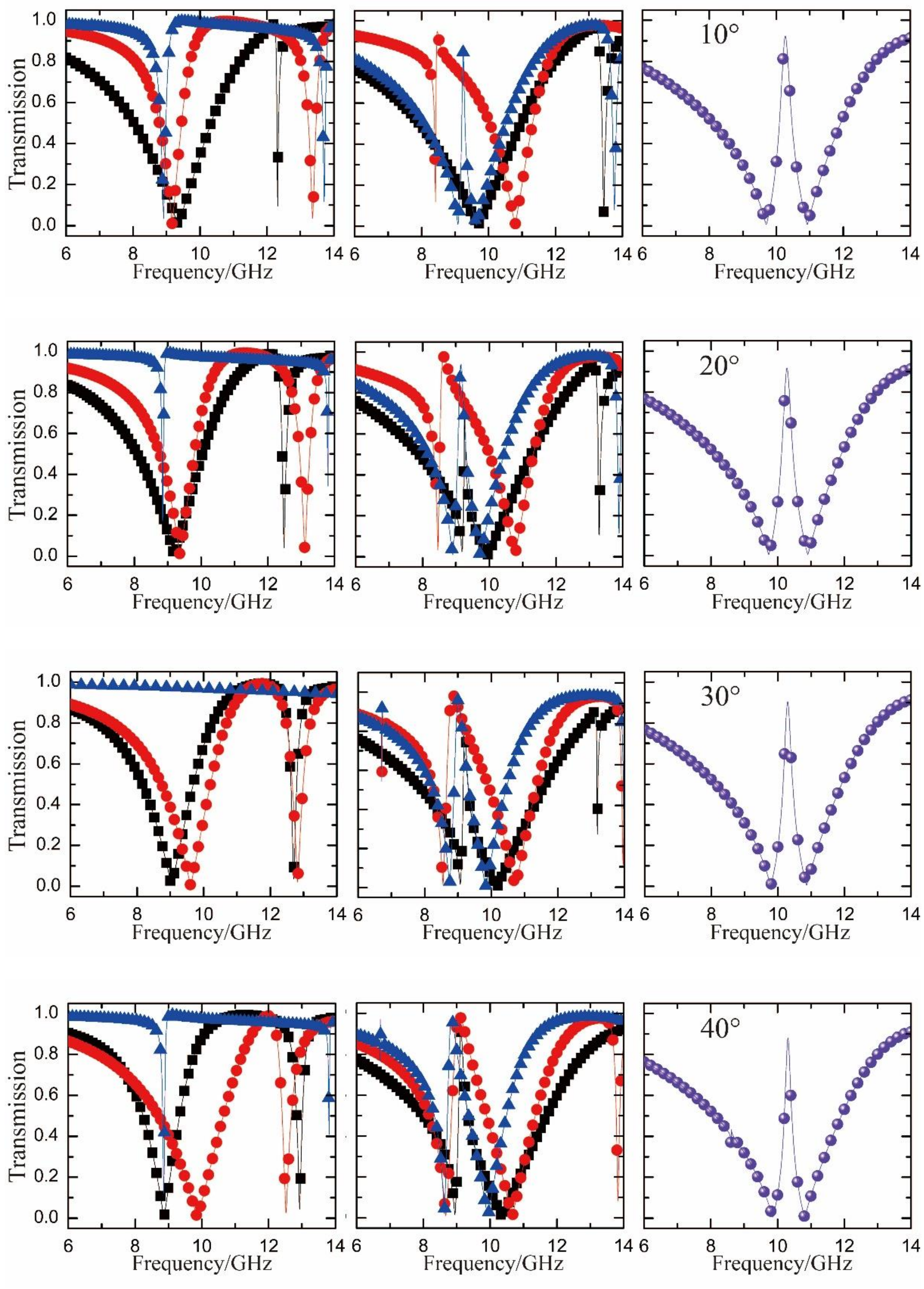

10°
20°
30°
40°
Transmission
Frequency/GHz

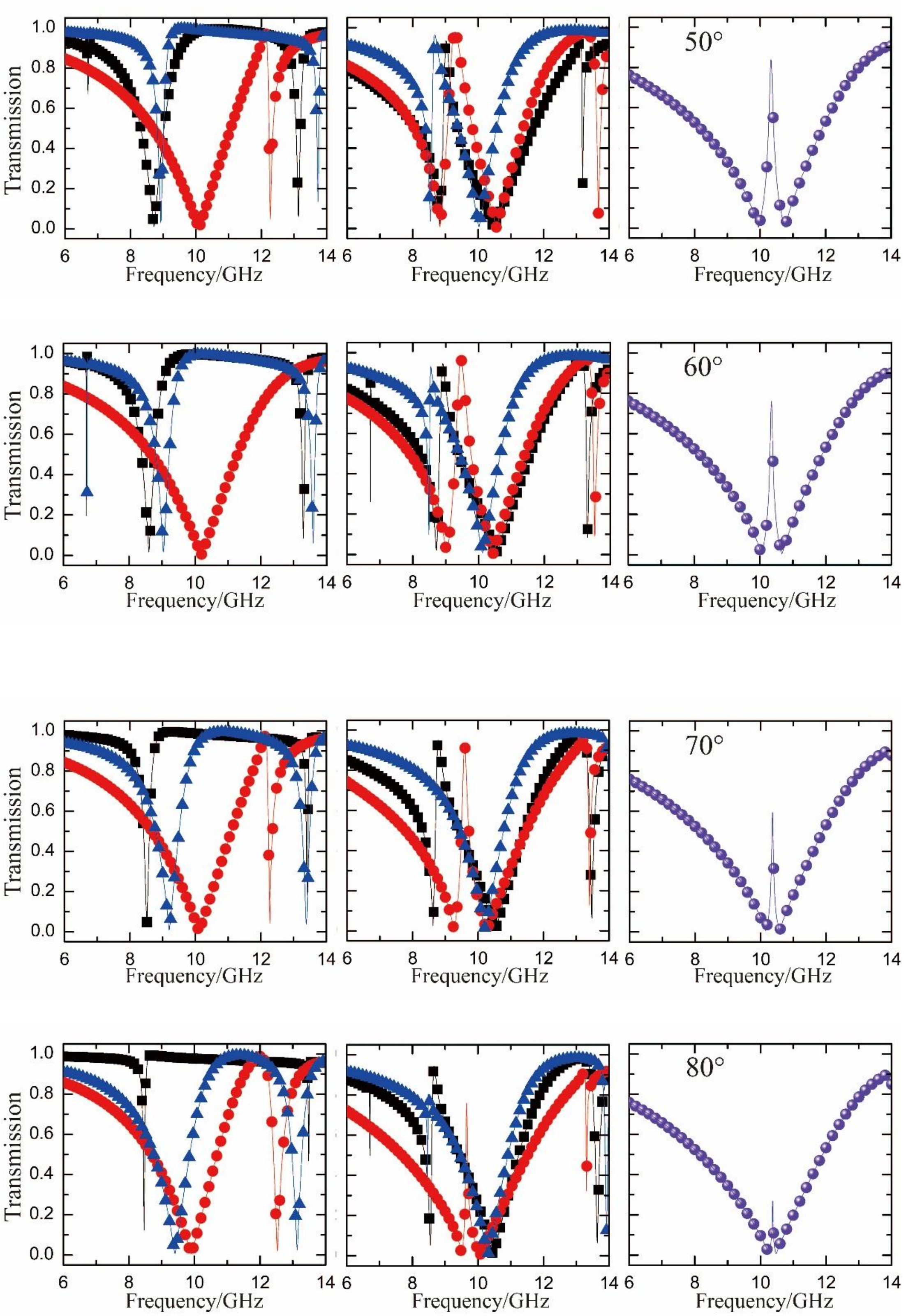
Transmission
Frequency/GHz
50°
60°
70°
80°

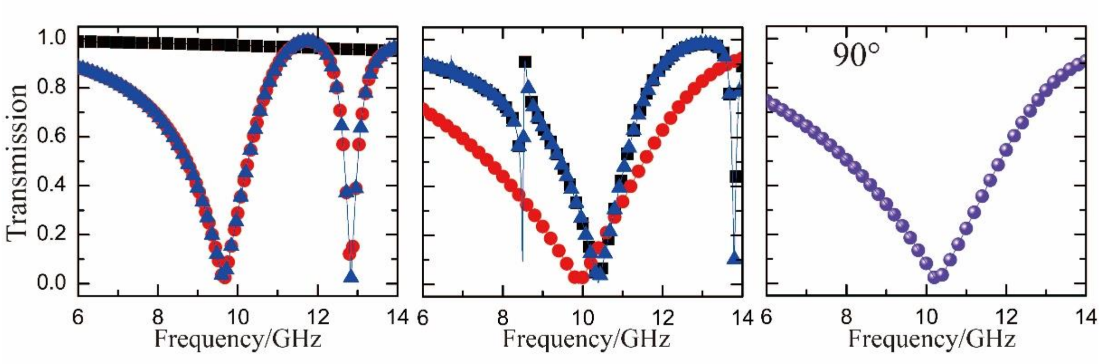

Figure S2. The simulated transmission spectra for the incident polarization angles change from 0 ° to 90 ° with steps of 10 °. (a) A single SSR, 0 ° arranged SRR (black), 60 ° arranged SRR (red), and 120 ° arranged SRR (blue).

For a single SRR1, the transmission curves show that the resonance is low-Q resonance to high-Q resonance and then non-resonance when the incident polarization angles change from 0 ° to 90 °, as shown in Fig. S2(a) black curves. Similarly, a single SRR2 exhibits the lowest Q resonance at the 60 ° incidence, and the transmission curves are identical for (30 °, 90 °), (40 °, 80 °), and (50 °, 70 °), showing nice symmetry with respect to 60 °. For a single SRR3, it exhibits a non-resonant transmission at 30 ° incidence, i.e. the incident electric field is parallel to the gap of SRR3. Moreover, the transmission curves show symmetric distribution with respect to the 30 ° incident polarization angle, and the transmission of SRR2 and SRR3 are identical for the 0 ° and 90 ° incidence. Fig. S2(a) shows that both high-Q and low-Q resonance can be tailored by rotating the orientation of the SRR. Despite showing different resonant frequency, two resonances with different Q-factor can still overlap and interfere destructively so that give rise to an interference-based transparent window, as shown in Fig. S2(b). In addition, two identical resonances with higher Q-factor overlap can form a lower Q-factor resonance, such as the resonant SRR combined by SRR2 and SRR3. Under 0 ° and 90 ° incidence, the combined SRR shows a higher-frequency lower-Q resonance (the red curves in Fig. S2(b)) compared to the resonance of SRR2 and SRR3 under 0 ° and 90 ° incidence (the red and blue curves in Fig. S2(a)). The decrease of Q-factor results from the combined SRR supports more bulky system so that increases the radiate loss. And compared to their single resonance, the resonant frequency of the SRR combined by SRR2 and SRR3 is blue-shift. The frequency shift origins from the fact that the combined SRR system can decrease the total capacitance and inductance. Because both of the capacitances and inductances are parallel connection by joining the SRRs together. Using the relation of $f \approx 1/2\pi\sqrt{LC}$, we know that the frequency goes up by decreasing the capacitance (C) and inductance (L). This can be used to better understand the blue-shift of the transparent window of the three SRRs system, as shown Fig. S2(c).

3. Evolution process of the electric/magnetic energy distribution

To clearly observe the evolution process of the near-field coupling between the SRRs, we simulated the electric/magnetic energy distributions at the transparent frequency with the incident polarization angles using steps of 10 °, as shown in Fig S3. The frequencies for every polarization angle can be found in **Table 1**, as shown in fourth part.

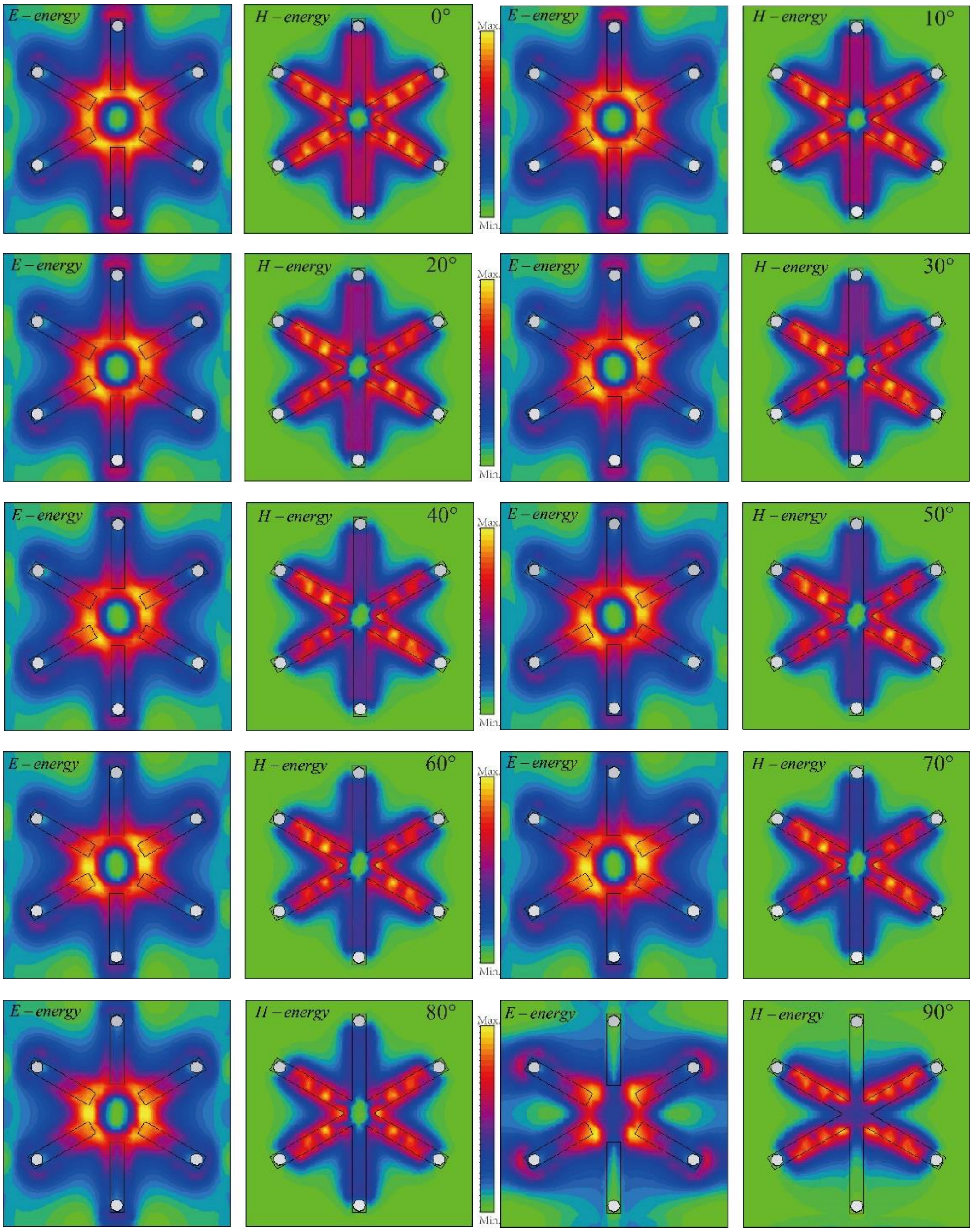

Figure S3. The evolution process of the electric energy and magnetic energy distribution by rotating the incident polarization angle from 0 ° to 90 °.

4. The parameter fitting using coupled oscillator model

By fitting the transmission curves, we use the parameters as shown in **Table 1**, and the variation tendencies of every parameters versus different incident polarization angles are plotted in Fig. S4. And the simulated, measured as well as the fitted results are plotted in Fig. S5, where it is clearly shown that the radiated loss of the bright mode (low-Q resonance) appears periodic property for the transparent modes. As we have illustrated, the bright mode will transit when from SRR1 to SRR2 when the incident polarization angle is rotated from 0 ° to 90 °. For the parameters of coupling strength Ω and detuning δ, we can clearly observe that these two parameters were decreasing when we changed the incident polarization angle, which implies that the Q-factor of the transparent

window will decrease as the coupling strength Ω and detuning δ decreasing. The transparent window will disappear when the coupling strength Ω deceases to zero. This makes sense because the coupling strength Ω characterize the interfering strength between bright mode and dark mode. Therefore, the transparent window will decease and disappear as the coupling strength deceasing from 1.268 GHz to 0. However, the detuning δ measure the frequency mismatch between bright mode and dark mode, which can be used to characterize the asymmetric degree of transparent window. For our proposed system, this property is clearly observed for the coupled oscillator mode, as shown in Figure S5 the fitting curves.

Table 1. The fitting parameters

Angle	γ_1/GHz	γ_2/GHz	ω_t/GHz	δ/GHz	Ω/GHz	g/GHz
**0 °**	2.454	0.14	10.264	0.584	1.268	1.1
**10 °**	2.294	0.14	10.272	0.576	1.224	1.05
**20 °**	1.952	0.14	10.28	0.552	1.176	1
**30 °**	1.511	0.14	10.296	0.512	1.06	0.88
**40 °**	1.818	0.14	10.312	0.456	0.96	0.96
**50 °**	2.233	0.14	10.328	0.384	0.8	1.04
**60 °**	2.464	0.14	10.352	0.312	0.632	1.1
**70 °**	2.265	0.14	10.36	0.208	0.424	1.05
**80 °**	1.866	0.14	10.376	0.12	0.224	0.96
**90 °**	5	0.14	--	0	0	1.5

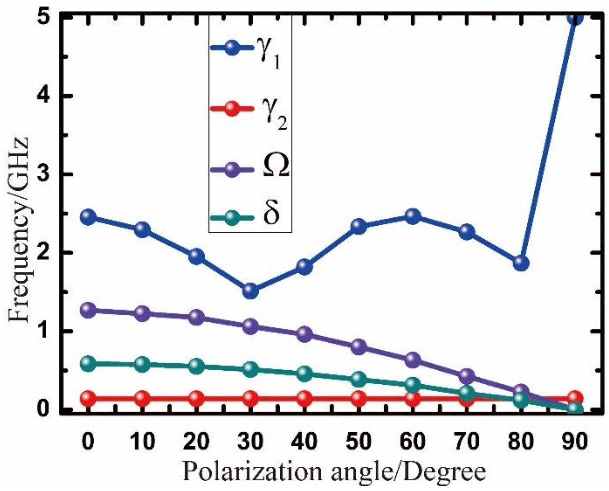

Figure S4. The polarization-dependent behavior of fitting parameters.

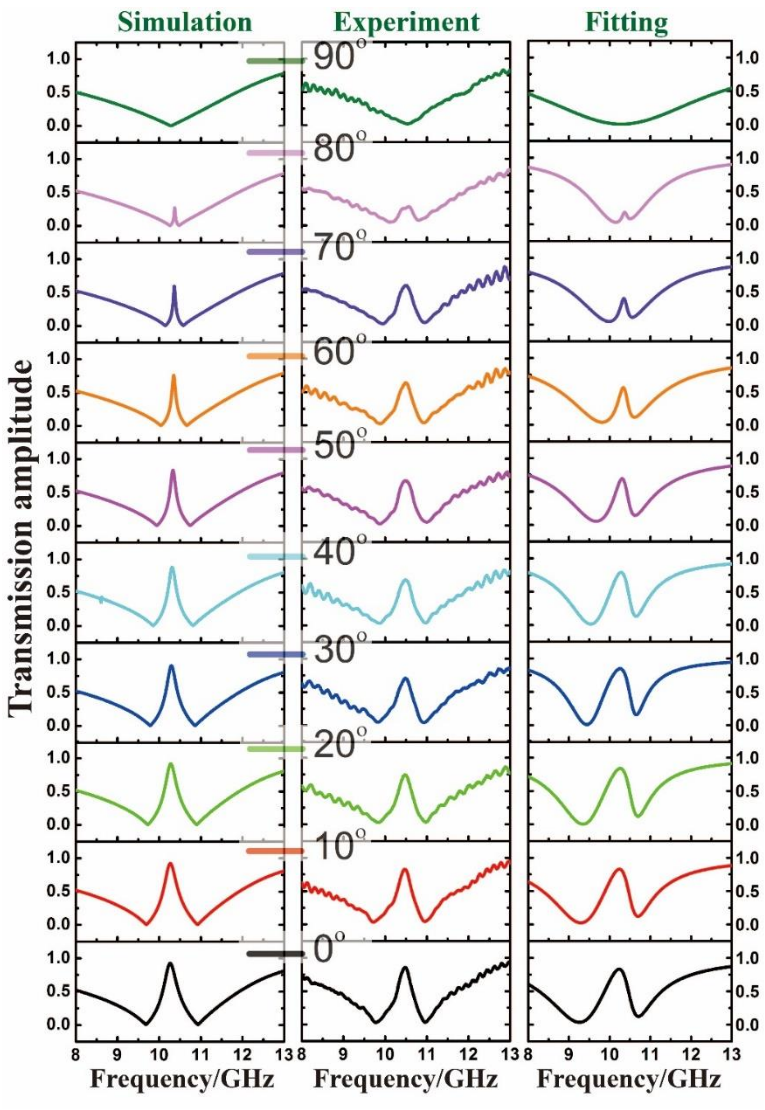

Figure S5. The simulated, measured as well as the fitted transmission spectra. The incident polarization vector angle is rotated with steps of 10 °.

5. Retrieved effective parameters

Figure S6 demonstrates the effective parameters, i.e., the effective refractive index (n_{eff}), permittivity (ε_{eff}), and permeability (μ_{eff}) as a function of frequency. The effective parameters are retrieved through S-parameter retrieval method [1]. As shown in Figure S6, all of the retrieved parameters show strong peaks for the imaginary parts around the transmission dips, which imply strong absorptions and scatterings at these transmission dips. From Figure S6 (a) and (c), it is also observed that the imaginary parts of refractive index and effective permittivity close to zero at the transparent range, which mean the very low loss of the proposed metamaterial at the transparent range.

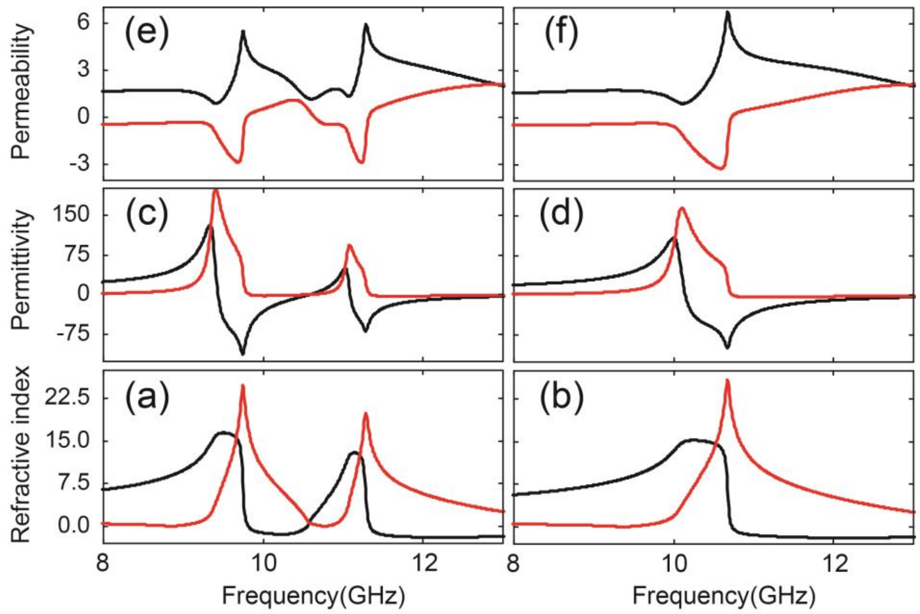

Figure S6. The retrieved parameters of refractive index (a) and (b), permittivity (c) and (d), and permeability (e) and (f) as a function of frequency, where the black curves are real parts and the red curves are imaginary parts. The incident polarization is 0 ° for (a), (c), (e) and 90 ° for (b), (d), (f).

References

[1] D. R. Smith, D. C. Vier, Th. Koschny, and C. M. Soukoulis, "Electromagnetic parameter retrieval from inhomogeneous metamaterials", PHY. REV. E 71, 036617 (2005).
